# Ticagrelor Versus Clopidogrel or Aspirin in Secondary Stroke Prevention: Systematic Review and Meta‐Analysis of Randomized Controlled Trials

**DOI:** 10.1155/srat/7411011

**Published:** 2026-05-09

**Authors:** Abdullah Faiz Zaihan, Nurul Ameera Huda Mohamad Hafiz, Yee Sheun Tong, Kaeshaelya Thiruchelvam, Chia Siang Kow

**Affiliations:** ^1^ Department of Pharmacy, Shah Alam Hospital, Shah Alam, Malaysia; ^2^ School of Pharmacy, IMU University, Kuala Lumpur, Malaysia, imu.edu.my; ^3^ School of Applied Sciences, University of Huddersfield, Huddersfield, UK, hud.ac.uk

**Keywords:** acute cerebrovascular syndrome, dual antiplatelet, pharmacogenetics, platelet aggregation inhibitor, secondary prevention, thienopyridine resistance

## Abstract

**Background:**

Ticagrelor, a direct‐acting P2Y_12_ receptor antagonist, has been proposed as an alternative to clopidogrel or aspirin for secondary prevention in patients with acute ischemic stroke or transient ischemic attack (TIA). However, its clinical utility remains uncertain due to variable trial findings and safety concerns. This study is aimed at evaluating the efficacy and safety of ticagrelor compared to clopidogrel or aspirin in reducing recurrent stroke and major bleeding in patients with noncardioembolic acute ischemic stroke or TIA.

**Methods:**

A systematic search of PubMed, Embase, CENTRAL, and Chinese databases was conducted to identify randomized controlled trials comparing ticagrelor with aspirin or clopidogrel in this population. The primary outcomes were recurrent stroke and major bleeding. Random‐effects meta‐analyses were performed, and heterogeneity was assessed using the *I* ^2^ statistic.

**Results:**

Six randomized controlled trials were included. Ticagrelor significantly reduced the risk of recurrent stroke compared to control (pooled OR: 0.78; 95% CI: 0.70–0.89; *I* ^2^ = 9*%*). No significant increase in major bleeding was observed (OR: 0.92; 95% CI: 0.66–1.28; *I* ^2^ = 0*%*). Risk of bias was low in two trials and raised some concerns in the remaining four due to open‐label designs or limited reporting.

**Conclusion:**

Ticagrelor offers superior efficacy over clopidogrel or aspirin in preventing recurrent stroke, with no excess risk of major bleeding. It may be particularly beneficial in patients with poor clopidogrel metabolism or high recurrence risk. Further research is needed to establish its long‐term safety, cost‐effectiveness, and optimal use in clinical practice.

**Summary**

• Meta‐analysis of six randomized controlled trials (RCTs) demonstrated that ticagrelor significantly lowered recurrent stroke events compared with aspirin or clopidogrel (pooled odds ratio [OR]: 0.78; 95% confidence interval [CI]: 0.70–0.89), with consistent benefit across diverse populations and stroke subtypes.
• Despite ticagrelor′s potent platelet inhibition, pooled data showed no statistically significant rise in major bleeding compared with control agents (OR: 0.92; 95% CI: 0.66–1.28), supporting its safety in the early poststroke setting.
• As a direct‐acting P2Y_12_ inhibitor, ticagrelor overcomes clopidogrel′s CYP2C19‐dependent variability, making it particularly valuable in populations with high prevalence of loss‐of‐function alleles, such as East Asians.

## 1. Introduction

Ischemic stroke and transient ischemic attack (TIA) remain major contributors to global morbidity and mortality. Survivors of an initial cerebrovascular event face a significantly elevated risk of early recurrence, particularly within the first 90 days, a period during which up to 10%–20% of patients may experience a recurrent stroke if not appropriately managed [1]. As such, the timely initiation of antiplatelet therapy is a cornerstone of secondary prevention in patients with noncardioembolic ischemic stroke or TIA.

Aspirin, a cyclooxygenase (COX) inhibitor, has traditionally served as the first‐line antiplatelet agent in this setting due to its accessibility and extensive evidence base. However, its antithrombotic effect is modest and variable. Several studies have demonstrated that aspirin monotherapy reduces the risk of recurrent stroke by only ~20%, leaving a considerable residual risk [2–5]. Additionally, aspirin resistance—a phenomenon observed in a substantial subset of patients—may further undermine its clinical efficacy. Moreover, the inhibition of thromboxane A_2_ alone may not provide sufficient platelet inhibition in the high‐risk poststroke state, particularly in the presence of large‐artery atherosclerosis or high platelet reactivity [6].

In contrast, clopidogrel, an ADP P2Y_12_ receptor antagonist, has shown superior efficacy compared to aspirin in certain subgroups and is widely used either as monotherapy or in combination with aspirin [7, 8]. However, clopidogrel is a prodrug requiring hepatic bioactivation via cytochrome P450 (particularly CYP2C19). This metabolic pathway is highly polymorphic, and loss‐of‐function CYP2C19 alleles are prevalent in many populations, particularly in East Asia. As a result, the antiplatelet efficacy of clopidogrel is inconsistent, and poor metabolizers may derive limited benefit, leading to higher rates of recurrent ischemic events [9].

Ticagrelor offers a potential solution to the pharmacogenomic limitations of clopidogrel. As a direct‐acting, reversible P2Y_12_ inhibitor, ticagrelor achieves rapid and potent platelet inhibition independent of metabolic activation, providing more consistent pharmacodynamic effects across genetic backgrounds [10]. It has been shown to be superior to clopidogrel in acute coronary syndrome and has been evaluated in recent RCTs for secondary stroke prevention, either as monotherapy or in combination with aspirin [11–13].

Despite these theoretical and pharmacological advantages, the clinical evidence for ticagrelor′s role in stroke prevention remains mixed. Some trials have demonstrated a reduction in recurrent ischemic events, while others have raised concerns about increased bleeding risk—particularly when used in dual antiplatelet therapy. In addition, prior trials have differed in terms of stroke severity, stroke mechanism (e.g., large‐vessel occlusion vs. minor stroke or TIA), and comparator agents, making it challenging to draw definitive conclusions [14].

This systematic review and meta‐analysis of RCTs is aimed at critically synthesizing available RCT evidence comparing ticagrelor to either clopidogrel or aspirin in patients with acute ischemic stroke or TIA, with a specific focus on two primary outcomes: recurrent stroke and bleeding risk. By consolidating efficacy and safety data across heterogeneous populations and study designs, we aim to provide evidence‐based clarity on whether ticagrelor offers meaningful clinical advantages in this high‐risk population.

## 2. Methods

### 2.1. Protocol and Reporting Standards

This systematic review and meta‐analysis was conducted in accordance with the Preferred Reporting Items for Systematic Reviews and Meta‐Analyses (PRISMA) 2020 guidelines [15]. The review protocol was prospectively developed and guided by the Cochrane Handbook for Systematic Reviews of Interventions [16]. The protocol was registered a priori in PROSPERO (ID: CRD420251054422).

### 2.2. Eligibility Criteria

#### 2.2.1. Types of Studies

We included RCTs that compared ticagrelor with either aspirin or clopidogrel, with or without combination therapy, in adult patients with acute ischemic stroke or TIA. Only full‐text articles published in peer‐reviewed journals were included. Observational studies, conference abstracts, reviews, editorials, protocols, and subanalyses of included trials were excluded.

#### 2.2.2. Types of Participants

Eligible studies enrolled adult patients (≥ 18 years) with a confirmed diagnosis of

• Acute ischemic stroke (any subtype, including minor stroke and large‐vessel occlusion), or
• TIA, as defined by each study′s protocol. Studies focusing on cardioembolic stroke or patients with atrial fibrillation were excluded to ensure homogeneity in the mechanism of stroke.

#### 2.2.3. Types of Interventions and Comparators

The intervention of interest was ticagrelor, administered either as

• Monotherapy, or
• In combination with aspirin.

The comparator arms included

• Aspirin monotherapy,
• Clopidogrel monotherapy, or
• Clopidogrel combined with aspirin.

#### 2.2.4. Types of Outcomes

We focused on the following primary outcomes:

1. Efficacy outcome: recurrent stroke (ischemic or hemorrhagic) at the longest available follow‐up (preferably 90 days)
2. Safety outcome: major bleeding events, as defined in each trial (e.g., PLATO, TIMI, or ECASS criteria)

### 2.3. Information Sources and Search Strategy

We systematically searched the following databases from inception to May 1, 2025:

• PubMed/MEDLINE
• Embase
• Cochrane Central Register of Controlled Trials (CENTRAL)
• Scopus

The search strategy combined MeSH terms and free‐text keywords for “ticagrelor,” “clopidogrel,” “aspirin,” “ischemic stroke,” and “transient ischemic attack.” No language restrictions were applied. The reference lists of included articles and relevant systematic reviews were also manually searched to identify additional studies.

### 2.4. Study Selection

All records were imported into Microsoft Excel for deduplication, and titles and abstracts were independently screened by two reviewers (AFZ and NAHMH). Full texts of potentially eligible articles were retrieved and assessed against the inclusion criteria. Discrepancies were resolved through discussion or consultation with a third reviewer (CSK). The study selection process was documented using a PRISMA 2020 flow diagram.

### 2.5. Data Extraction

A standardized data extraction form was developed and pilot‐tested. Two reviewers (AFZ and NAHMH) independently extracted the following information:

• Study characteristics: author, year, country, and design
• Population characteristics: inclusion/exclusion criteria, sample size, mean/median age, and baseline NIHSS or mRS
• Intervention details: dose, timing, and duration of ticagrelor
• Comparator details: type of control (aspirin and clopidogrel), dose, and schedule
• Outcome data: number of events and total participants for recurrent stroke and major bleeding
• Follow‐up duration

Disagreements in data extraction were resolved by consensus or adjudication by a third reviewer (YST).

### 2.6. Risk‐of‐Bias Assessment

Risk of bias was assessed independently by two reviewers (CSK and AFZ) using the Cochrane Risk of Bias 2.0 (RoB 2) tool [17]. The following five domains were evaluated:

1. Bias arising from the randomization process
2. Bias due to deviations from intended interventions
3. Bias due to missing outcome data
4. Bias in the measurement of the outcome
5. Bias in the selection of the reported result

Each domain in the Risk of Bias 2.0 tool was assessed as having either “Low risk of bias,” “Some concerns,” or “High risk of bias.” To determine the overall risk of bias for each study, the judgments from the five individual domains were integrated following Cochrane′s prespecified algorithm:

• A study was judged to have “Low risk of bias overall” if all domains were assessed as low risk.
• A study was rated as having “Some concerns overall” if at least one domain raised some concerns, but none were judged to be high risk.
• A study was considered to have “High risk of bias overall” if one or more domains were rated as high risk.

Discrepancies were resolved by discussion or third‐party arbitration (KT).

### 2.7. Data Synthesis and Statistical Analysis

Meta‐analyses were performed using MetaXL Version 5.3 (EpiGear International, Sunrise Beach, Australia), an Excel‐based tool for meta‐analysis that implements both fixed‐effect and random‐effects models. The random‐effects model using the DerSimonian and Laird method was applied as the default approach, accounting for between‐study heterogeneity in effect sizes.

Pooled ORs with corresponding 95% CIs were calculated for dichotomous outcomes, namely, recurrent stroke and bleeding events.

Statistical heterogeneity across studies was assessed using

• The *I* ^2^ statistic, where values of 25%, 50%, and 75% indicated low, moderate, and high heterogeneity, respectively
• The Cochran *Q* test, with a *p* value < 0.10 considered indicative of significant heterogeneity

All statistical tests were two‐sided, and results were considered statistically significant at *p* < 0.05, unless stated otherwise.

## 3. Results

### 3.1. Study Selection and Characteristics

**Figure 1:**
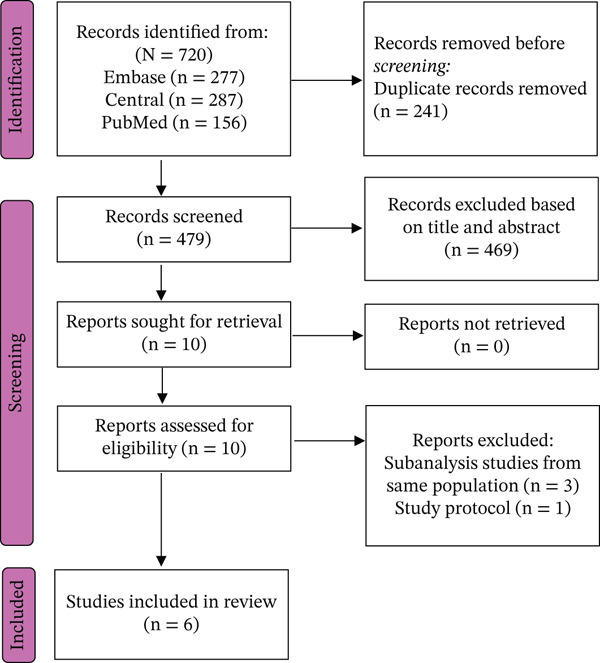
PRISMA 2020 flow diagram of study selection.

A total of 720 records were identified through database searches. After removal of 241 duplicates and screening of 479 titles and abstracts, 10 full‐text articles were assessed for eligibility. Ultimately, six RCTs [18–23] were included in the meta‐analysis. The PRISMA flow diagram detailing the study selection process is shown in Figure 1.

Table 1 summarizes the key characteristics of the included trials. The studies were conducted in diverse settings, including China, Egypt, and multiple international regions, and varied in design, with three employing a double‐blind randomized approach and three utilizing open‐label or single‐blind designs. Sample sizes ranged from 169 to over 13,000 participants.

**Table 1:** Characteristics of included randomized controlled trials comparing ticagrelor with clopidogrel or aspirin in patients with ischemic stroke or TIA.

Study	Country	Design	Sample size (ticagrelor vs. control)	Study population	Mean/median age (ticagrelor vs. control)	Follow‐up	Ticagrelor regimen	Control regimen	Recurrent stroke (*n*/*N*; % [ticagrelor vs. control])	Major bleeding	Risk of bias
Wang et al. [20]	China	Randomized, double‐blind, placebo‐controlled trial	3205 vs. 3207	Minor stroke or TIA with CYP2C19 loss‐of‐function alleles	63.0 vs. 63.2	3 months	Ticagrelor + aspirin (90 mg BID, +75–300 mg QD)	Clopidogrel + aspirin (300 mg LD → 75 mg QD)	191/3205; 6.0% vs. 243/3207; 7.6%	9/3205; 0.3% vs. 11/3207; 0.3%	Low
Wang et al. [22]	China	Randomized, open‐label, controlled trial	336 vs. 339	Minor stroke or TIA	61.1 vs. 60.5	3 months	Ticagrelor 180 mg LD → 90 mg BID + aspirin	Clopidogrel 300 mg LD → 75 mg OD + aspirin	21/336; 6.2% vs. 30/339; 8.8%	5/336; 1.5% vs. 4/339; 1.2%	Some concerns
Zeinhom et al. [21]	Egypt	Randomized, open‐label, controlled trial	85 vs. 84	First‐ever ischemic stroke	59.4 vs. 58.9	3 months	Ticagrelor 180 mg LD → 90 mg BID	Aspirin 300 mg LD → 75 mg QD	4/85; 4.7% vs. 5/84; 6.0%	1/85; 1.2% vs. 2/84; 2.4%	Some concerns
Zeinhom et al. [19]	Egypt	Randomized, single‐blind, controlled trial	290 vs. 290	Large‐vessel ischemic stroke	60.0 vs. 60.0	3 months	Ticagrelor 180 mg LD → 90 mg BID	Clopidogrel 300 mg LD → 75 mg QD	30/290; 10.3% vs. 49/290; 16.9%	10/290; 3.4% vs. 9/290; 3.1%	Some concerns
Johnston et al. [18]	Multicountry (various regions)	Randomized, double‐blind, controlled trial	6589 vs. 6610	Acute ischemic stroke or high‐risk TIA	64.0 vs. 64.0	3 months	Ticagrelor 90 mg BID	Aspirin 100 mg QD	385/6589; 5.8% vs. 441/6610; 6.7%	31/6589; 0.5% vs. 38/6610; 0.6%	Low
Ahmed et al. [23]	Egypt	Randomized, single‐blind, controlled trial	450 vs. 450	First‐ever, noncardioembolic moderate or moderate‐to‐severe acute ischemic stroke	Not reported	3 months	Ticagrelor 180 mg LD → 90 mg BID	Clopidogrel 300 mg LD → 75 mg QD	39/450; 8.7% vs. 62/450; 13.8%	12/450; 2.7% vs. 10/450; 2.7%	Some concerns

Abbreviations: BID, twice daily; LD, loading dose; *n*/*N*, number of events/total number of patients; QD, once daily; TIA, transient ischemic attack.

The study populations included patients with minor stroke or TIA [20, 22], first‐ever ischemic stroke [21], large‐vessel ischemic stroke [19], and noncardioembolic acute stroke or high‐risk TIA [18, 23]. The mean or median ages were comparable between treatment groups across all trials, ranging from 59.4 to 64.0 years in the ticagrelor arms and 58.9 to 64.0 years in the control arms. All studies had a follow‐up period of 3 months.

Ticagrelor regimens varied slightly, ranging from 90 mg BID monotherapy in four studies [18, 19, 21, 23] to dual antiplatelet therapy (ticagrelor plus aspirin) in two studies [20, 22]. Comparators included clopidogrel plus aspirin, clopidogrel monotherapy, or aspirin monotherapy.

Risk of bias was assessed using the Cochrane RoB 2.0 tool. Two trials [18, 20] were rated at low risk of bias, while the remaining four [19, 21–23] were rated as having some concerns, primarily due to open‐label or single‐blind designs and/or limited reporting on allocation concealment.

### 3.2. Recurrent Stroke

**Figure 2:**
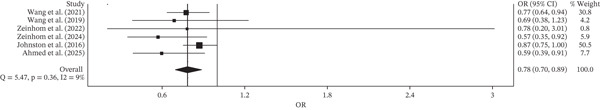
Forest plot of the effect of ticagrelor versus aspirin or clopidogrel on recurrent stroke.

All six studies reported data on recurrent stroke events. Across the trials, a total of 10,955 patients received ticagrelor and 10,980 received a comparator (aspirin or clopidogrel). Meta‐analysis using a random‐effects model showed that ticagrelor significantly reduced the risk of recurrent stroke compared to either aspirin or clopidogrel (pooled OR: 0.78; 95% CI: 0.70–0.89) (Figure 2). Heterogeneity across the studies was negligible (*Q* = 5.47, *p* = 0.36, *I* ^2^ = 9*%*), indicating a high level of consistency in the treatment effect across diverse populations and trial designs.

### 3.3. Major Bleeding

**Figure 3:**
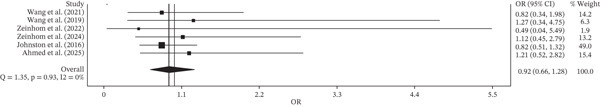
Forest plot of the effect of ticagrelor versus aspirin or clopidogrel on major bleeding events.

All five included trials also reported major bleeding events as defined by study‐specific criteria (e.g., PLATO or ECASS classification). Across the trials, a total of 10,955 patients received ticagrelor and 10,980 received a comparator (aspirin or clopidogrel). The pooled estimate showed no statistically significant increase in major bleeding associated with ticagrelor compared to control (OR: 0.92; 95% CI: 0.66–1.28) (Figure 3). The analysis demonstrated no evidence of heterogeneity (*Q* = 1.35, *p* = 0.93, *I* ^2^ = 0*%*).

## 4. Discussion

This meta‐analysis of five RCTs provides compelling evidence that ticagrelor offers superior efficacy to either clopidogrel or aspirin in preventing recurrent stroke in patients with acute ischemic stroke or TIA. The benefit was consistent across trials, with a statistically significant reduction in recurrent stroke and negligible between‐study heterogeneity, underscoring the reliability and generalizability of the pooled findings. These results affirm the evolving role of ticagrelor as a viable antiplatelet agent in cerebrovascular disease, particularly in settings where clopidogrel resistance or suboptimal platelet inhibition may compromise outcomes.

The pharmacological profile of ticagrelor offers a mechanistic explanation for its observed superiority. Unlike clopidogrel, which requires hepatic biotransformation via the CYP2C19 pathway, ticagrelor is an active, reversible P2Y_12_ receptor antagonist that achieves more rapid, potent, and consistent platelet inhibition [9, 10]. This advantage is especially relevant in East Asian populations, where the prevalence of CYP2C19 loss‐of‐function alleles can reach 50%–60%, leading to subtherapeutic platelet inhibition with clopidogrel [24, 25]. In CHANCE‐2, which specifically enrolled patients with these alleles, ticagrelor′s benefit was particularly pronounced [17]. In CHANCE‐2, ticagrelor demonstrated a clearer reduction in recurrent ischemic events compared with clopidogrel [17], highlighting the importance of pharmacogenomic factors in modulating treatment response. In contrast, trials conducted in nongenotyped and multiregional populations, including those enrolling patients from the Middle East and Africa, showed more modest but directionally consistent benefits, suggesting that population‐level differences in genetic background may partially contribute to variability in treatment effect. Moreover, by acting through a different pathway than aspirin—targeting ADP‐mediated platelet activation rather than thromboxane A_2_—ticagrelor may also offer enhanced protection in patients whose stroke pathophysiology involves platelet hyperreactivity not fully suppressed by COX‐1 inhibition [26].

Notably, this efficacy did not come at the expense of increased major bleeding. While ticagrelor′s potent antiplatelet activity raised theoretical concerns about hemorrhagic complications, particularly when used in combination with aspirin, our analysis did not detect a significant increase in major bleeding events. The bleeding rates remained low across all trials, with overlapping CIs between the ticagrelor and control groups. This observation remained consistent even in studies enrolling patients with moderate or moderate‐to‐severe ischemic stroke, in whom baseline risks of hemorrhagic transformation are inherently higher due to larger infarct size and more severe neurological deficits. This suggests that in carefully selected patients, the bleeding risk is manageable and does not offset the gains in stroke prevention. However, this finding must be interpreted with clinical nuance, as even a modest absolute increase in bleeding could be clinically relevant in frail patients, those with a history of gastrointestinal bleeding, or those on concurrent anticoagulant therapy.

An important strength of this review is the inclusion of diverse study populations and stroke subtypes, from minor stroke and TIA to moderate‐to‐severe ischemic strokes due to large‐vessel occlusion [18–22]. While earlier trials predominantly focused on minor stroke or TIA, more recent evidence, including the TICA‐CLOP trial [26], expanded evaluation to patients with first‐ever noncardioembolic moderate and moderate‐to‐severe ischemic stroke, thereby broadening the clinical applicability of ticagrelor. The observed consistency in ticagrelor′s benefit across these different clinical scenarios strengthens the external validity of the findings. Moreover, the studies spanned multiple regions—Asia, the Middle East, and global multicenter trials—reinforcing ticagrelor′s applicability across ethnic and geographic boundaries. The consistency of treatment effect despite variations in aspirin dosing, clopidogrel use, and stroke mechanisms suggests that the core benefit of ticagrelor is robust across a wide clinical spectrum.

Despite these strengths, several limitations warrant discussion. First, the follow‐up period in all included trials was limited to 3 months, reflecting the early poststroke period when recurrence risk is highest but leaving unanswered questions about the long‐term efficacy and safety of ticagrelor. The absence of longer term data precludes definitive conclusions about sustained benefit or late‐emerging bleeding risks. Second, while all trials reported adjudicated outcomes, three of the five studies were open‐label or single‐blinded and, thus, were rated as having “some concerns” in risk of bias due to the potential for performance or detection bias. Although endpoint adjudication was blinded, awareness of treatment assignment may have influenced clinician behavior or patient‐reported events. Third, the meta‐analysis did not stratify findings based on stroke mechanism (e.g., large artery atherosclerosis vs. small vessel disease), prior antiplatelet use, or pharmacogenomic status due to the small number of included studies and limited availability of subgroup‐level data in the published reports, all of which may influence individual response to therapy. Lastly, publication bias could not be formally assessed due to the small number of included trials per outcome.

From a clinical implementation standpoint, the findings support greater consideration of ticagrelor as an alternative to clopidogrel or aspirin, particularly in patients at high risk for recurrent stroke or with known clopidogrel resistance. However, clopidogrel retains important practical advantages, including lower cost, once‐daily dosing, and wider availability as a generic formulation, which may favor adherence and accessibility in routine practice. Ticagrelor is significantly more expensive than generic antiplatelet agents, which may limit its use in resource‐constrained settings or health systems with restrictive formularies. A pragmatic approach may involve selective use in high‐risk subgroups, such as those with CYP2C19 loss‐of‐function alleles, patients with large‐vessel occlusion or moderate‐to‐severe stroke, or early recurrence despite standard therapy. In centers with genotyping capabilities, a personalized, pharmacogenomics‐guided strategy may offer an optimal balance between efficacy, safety, and cost‐effectiveness.

These findings also open several promising avenues for future research. One critical area is the long‐term safety and efficacy of ticagrelor beyond the 90‐day period evaluated in current trials. Given the persistent risk of recurrent stroke over time, studies exploring extended‐duration therapy, optimal de‐escalation strategies, or transition to other agents are warranted. Furthermore, head‐to‐head comparisons of ticagrelor with newer antiplatelet regimens, including cilostazol or low‐dose rivaroxaban combinations, could better position ticagrelor within the evolving antithrombotic landscape. Another important direction is pharmacogenomic integration into clinical decision‐making, particularly randomized trials evaluating genotype‐guided antiplatelet therapy versus standard care. In parallel, biomarker‐driven risk stratification—for both ischemic and bleeding events—may help refine patient selection and individualize treatment duration. Lastly, real‐world effectiveness studies and cost‐effectiveness analyses in different healthcare systems are essential to assess ticagrelor′s feasibility and impact outside the trial setting, especially in low‐ and middle‐income countries where stroke burden is highest and resources are limited.

## 5. Conclusion

This systematic review and meta‐analysis demonstrates that ticagrelor significantly reduces the risk of recurrent stroke compared to aspirin or clopidogrel in patients with acute ischemic stroke or TIA, without a corresponding increase in major bleeding. The consistent direction of benefit across diverse populations and stroke subtypes reinforces ticagrelor′s potential as an effective alternative antiplatelet agent, particularly in settings where clopidogrel resistance or high risk of recurrence are concerns. While the findings are encouraging, they are based on short‐term follow‐up and a limited number of trials. Future research should focus on longer term outcomes, patient selection strategies, and cost‐effectiveness to better define ticagrelor′s role in clinical practice.

## Author Contributions

**Abdullah Faiz Zaihan:** conceptualization; roles/writing—original draft, review, and editing; and final approval of the version to be published. **Chia Siang Kow:** conceptualization; data analysis; roles/writing—original draft, review, and editing; and final approval of the version to be published. **Nurul Ameera Huda Mohamad Hafiz:** roles/writing—original draft, review, and editing—and final approval of the version to be published. **Yee Sheun Tong:** roles/writing—original draft, review, and editing—and final approval of the version to be published. **Kaeshaelya Thiruchelvam:** roles/writing—original draft, review, and editing—and final approval of the version to be published.

## Funding

No funding was received for this manuscript.

## Disclosure

This work was previously presented as a conference abstract and published in the *British Journal of Clinical Pharmacology* (https://bpspubs.onlinelibrary.wiley.com/doi/10.1002/bcp.70349). The current manuscript represents the full version of the work.

## Ethics Statement

The authors have nothing to report.

## Consent

The authors have nothing to report.

## Conflicts of Interest

The authors declare no conflicts of interest.

## Data Availability

Data will be made available upon reasonable request.
